# Bronchopulmonary Dysplasia: Chronic Lung Disease of Infancy and Long-Term Pulmonary Outcomes

**DOI:** 10.3390/jcm6010004

**Published:** 2017-01-06

**Authors:** Lauren M. Davidson, Sara K. Berkelhamer

**Affiliations:** Department of Pediatrics, University at Buffalo SUNY, Buffalo, NY 14228, USA; lmdavids@buffalo.edu

**Keywords:** bronchopulmonary dysplasia, neonatal lung injury, chronic lung disease of prematurity, long-term outcomes

## Abstract

Bronchopulmonary dysplasia (BPD) is a chronic lung disease most commonly seen in premature infants who required mechanical ventilation and oxygen therapy for acute respiratory distress. While advances in neonatal care have resulted in improved survival rates of premature infants, limited progress has been made in reducing rates of BPD. Lack of progress may in part be attributed to the limited therapeutic options available for prevention and treatment of BPD. Several lung-protective strategies have been shown to reduce risks, including use of non-invasive support, as well as early extubation and volume ventilation when intubation is required. These approaches, along with optimal nutrition and medical therapy, decrease risk of BPD; however, impacts on long-term outcomes are poorly defined. Characterization of late outcomes remain a challenge as rapid advances in medical management result in current adult BPD survivors representing outdated neonatal care. While pulmonary disease improves with growth, long-term follow-up studies raise concerns for persistent pulmonary dysfunction; asthma-like symptoms and exercise intolerance in young adults after BPD. Abnormal ventilatory responses and pulmonary hypertension can further complicate disease. These pulmonary morbidities, combined with environmental and infectious exposures, may result in significant long-term pulmonary sequalae and represent a growing burden on health systems. Additional longitudinal studies are needed to determine outcomes beyond the second decade, and define risk factors and optimal treatment for late sequalae of disease.

## 1. Introduction

Bronchopulmonary dysplasia (BPD) is a chronic lung disease most commonly seen in premature infants who required mechanical ventilation and oxygen therapy for acute respiratory distress but can also occur in neonates that had a less severe respiratory course [1,2,3]. BPD was first reported in 1967 by Northway et al., in a group of premature infants who developed chronic pulmonary disease after receiving ventilation and supraphysiologic oxygen for respiratory distress syndrome [1]. While advances in neonatal care have resulted in improved survival rates of premature infants, limited progress has been made in reducing rates of BPD. However, several series have identified that infants suffer less severe disease with decreased risk of mortality than what was originally observed and described by Northway [4,5]. Introduction of antenatal steroids, postnatal surfactant, modern respiratory care and improved nutrition has resulted in milder pulmonary sequelae with less lung fibrosis but histopathologic evidence of arrested lung development, referred to by some as the “new BPD” [4,6,7,8]. Despite widespread efforts to protect against injury of the vulnerable premature lung, BPD remains the most frequent adverse outcome for infants born less than 30 weeks gestational age and the most common chronic lung disease in infancy [9]. Further studies have identified that BPD and prematurity have long-term impacts on pulmonary function and may increase risk of late pulmonary morbidity, raising concerns that newborn intensive care unit (NICU) survivors represent an emerging burden and future challenge for health systems and adult providers.

### 1.1. Prevalence of Bronchopulmonary Dysplasia (BPD)

The incidence of BPD in surviving infants less than or equal to 28 weeks gestational age has been relatively stable at approximately 40% over the last few decades [5,10,11,12]. At present, this results in an estimated 10,000–15,000 new cases annually in the US alone [13,14]. While several studies suggest increasing rates of infants surviving with BPD, changes in definitions and variable approaches to the use of oxygen therapy both influences and complicates interpretation of the historical data [5,15]. Although routine use of antenatal steroids with threatened preterm birth as well as surfactant administration with respiratory distress syndrome have greatly impacted survival of premature infants, these advances do not appear to have translated into decreasing rates of BPD [16,17]. Progress is hindered by the lack of effective therapies to prevent neonatal lung injury and chronic disease. Current lung protective strategies being encouraged include use of volume-targeted as well as non-invasive ventilation, permissive hypercapnia, and targeted use of steroids along with adjunct medical therapies including caffeine, and vitamin A.

### 1.2. Definition of BPD

Criteria to define BPD have historically lacked uniformity. The earliest clinical definition of BPD was limited to oxygen requirement at 28 days with consistent radiologic changes. These were originally modified to include continuing need for oxygen therapy at 36 weeks corrected gestational age (CGA). However, this definition inadequately addresses highly variable clinical practices as well as the wide range of disease, leading to further modification to include a severity assessment at 36 weeks gestational age [3,18]. The definition now takes into account total duration of oxygen supplementation, positive pressure requirements and gestational age, in addition to oxygen dependency at 36 weeks post menstrual age (PMA). (Table 1) This distinction has helped to identify that severity of BPD influences both pulmonary and neurodevelopmental outcomes as well as risk of mortality [19]. However, numerous limitations remain as the system fails to adequately classify infants with respect to airway issues (including tracheal or bronchomalacia and/or reactive airway disease) and pulmonary vascular disease [20]. In addition, contemporary management, including use of high-flow nasal cannula, is not addressed and can result in misclassification.

At greatest risk of poor long-term outcome are the approximately 25% of infants with BPD who are identified to have elevated pulmonary pressures or BPD-associated pulmonary hypertension (PH), resulting in mortality rates as high as 14%–38% with one series noting only 25% survival at 2–3 years of age with severe BPD-associated PH [21,22,23,24].

As the population of NICU survivors grow, long-term manifestations of chronic lung injury with BPD is likely to represent a greater burden to health systems. This paper seeks to review the pathophysiology of BPD, recent management strategies and what is currently known about long-term pulmonary outcomes in survivors.

## 2. Pathophysiology of BPD

**Figure 1:**
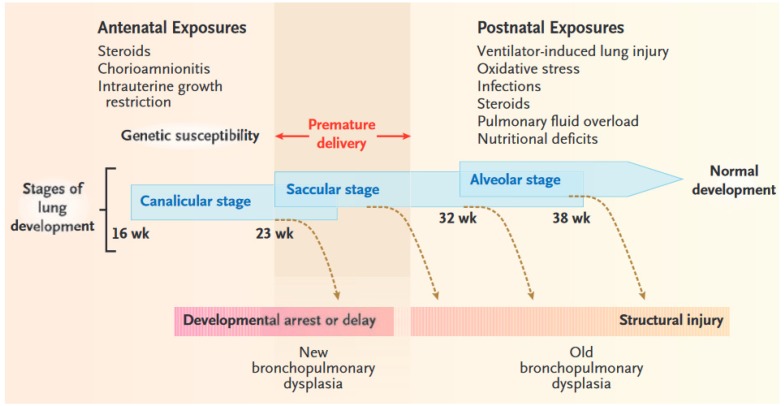
Stages of Lung Development, Potentially Damaging Factors, and Types of Lung Injury. In premature newborns, the lungs are often exposed to several sources of injury, both before and after birth. These exposures, along with genetic susceptibility to problematic lung development, can cause direct airway and parenchymal damage and induce a deviation from the normal developmental path. Depending on the timing and extent of the exposures, lung injury may range from early developmental arrest (new bronchopulmonary dysplasia) to structural damage of a relatively immature lung (old bronchopulmonary dysplasia). Premature infants born at a gestational age of 23 to 30 weeks (shaded region)—during the canalicular and saccular stages of lung development—are at the greatest risk for bronchopulmonary dysplasia. From Eugenio Baraldi, M.D.; Marco Filippone, M.D. Chronic Lung Disease after Premature Birth. *N. Engl. J. Med.* 2007, 357, 1946–1955. Copyright © 2007 Massachusetts Medical Society. Reprinted with permission from Massachusetts Medical Society.

The phenotype seen with BPD is the end result of a complex multifactorial process in which various pre- and postnatal factors compromise normal development in the immature lung. (Figure 1) The specific timing and duration of exposures influences the pattern of pulmonary damage which may occur [25]. Notably, the prevalence of BPD in mechanically ventilated infants is inversely related to gestational age and birth weight, supporting that incomplete development of the lungs or injury during a critical window of lung development influence the development of BPD [4]. In addition to prematurity, several other factors can contribute to disruption of alveolar growth and pulmonary vascular development including but not limited to mechanical ventilation, oxygen toxicity, pre- and postnatal infection, inflammation, and growth restriction or nutritional deficits. Genetic predisposition is recognized to further modify the risk of disease.

### 2.1. Mechanical Trauma

BPD occurs almost exclusively in preterm infants that have received positive pressure ventilation suggesting that mechanical lung over-distension and alveolar stretch play a critical role in the pathogenesis of BPD. Ineffective pulmonary mechanics results in need for ventilatory assistance at birth. Recent data suggests that 65% of preterm infants born at 22 to 28 weeks gestational age are intubated in the delivery room, which has decreased since 1993 when 80% of this population was intubated immediately following birth [26]. The premature lung is often difficult to ventilate due to surfactant deficiency resulting in decreased compliance and challenges maintaining functional residual capacity (FRC) [27,28]. Surfactant deficiency further contributes to non-uniform expansion of the lung with areas of focal over-distension and atelectasis [27,29]. Positive pressure and excess volume delivered via assisted ventilation can cause injury to the immature lung by further over-inflation of alveoli, leading to cellular injury, inflammation and reactive oxygen species (ROS) generation, thereby potentially amplifying preexisting injury associated with prenatal inflammation [9,30].

### 2.2. Oxygen Toxicity

Studies in numerous animal models have identified that exposure to supraphysiologic oxygen alone induces a phenotype comparable to that seen with BPD, including compromised alveolar development and pulmonary vascular remodeling [31]. Clinical studies parallel these findings with some evidence of decrease in lung inflammation and rates of BPD with restricted use of oxygen or lower saturation targets [32,33]. In addition, while endotracheal administration of recombinant superoxide dismutase to premature infants did not reduce rates of BPD, long-term pulmonary outcomes were improved supporting the critical contribution of oxidative injury [34]. Supraphysiologic oxygen results in increased mitochondrial ROS generation with unique susceptibility to oxidative stress and alveolar cell injury in the developing lung, in part attributable to antioxidant deficiencies and immature defenses [35,36,37].

Animal models suggest that even brief exposures to high concentrations of oxygen can result in long-term morphologic and functional changes in the lung [38]. In addition, a critical window of susceptibility to oxidative lung injury may exist in the immature lung [39]. Supporting these concerns are clinical data suggesting that even brief exposure to supraphysiologic oxygen during resuscitation increases the risk of BPD [33], and that prolonged evidence of oxidative stress can be identified in exhaled breath condensate of adolescents born preterm [40].

### 2.3. Infection and Inflammation

Controversy exists regarding the contribution of chorioamnionitis and prenatal inflammation to the risk of developing BPD [41]. Clinical and experimental studies have suggested that chorioamnionitis induces early lung maturation with increased surfactant production and decreased risk of RDS [42,43]. However, studies have also raised concerns for associated lung injury and decreased alveolarization. Administration of *Escherichia Coli* endotoxin to pregnant ewes resulted in amplified inflammation with ventilation of the exposed preterm lambs including evidence of cellular apoptosis and compromised alveolar development [44,45]. While several clinical studies have reported an association between chorioamnionitis and BPD, a meta-analysis including 59 studies and over 15,000 infants suggested that limited association between chorioamnionitis and BPD existed when adjustments were made for gestational age [43]. This paper also raised concerns for publication bias and concluded that chorioamnionitis cannot be definitively considered a risk for BPD [41]. Controversy exists as variable definitions have been used to classify chorioamnionitis and the term itself may represent a range of pathology. Recent analysis of data from a 25-year cohort of over 1600 very-low-birth weight infants concluded that sepsis, but not chorioamnionitis, increased risks of developing moderate or severe BPD [46].

Less controversy exists regarding the contribution of postnatal inflammation or nosocomial infection to the increased risk of developing BPD [47,48]. Novitsky et al. identified that premature infants with BPD were more likely to receive prolonged courses of antibiotics in the first week of life and to have evidence of resistant gram-negative bacilli in their endotracheal tube [49]. This data raises concerns that the presence of resistant organisms may result in more severe infection, advocating for judicious use of prophylactic or prolonged antibiotics in premature infants at risk. Non-infectious exposures, including oxygen and mechanical ventilation, cause further injury to the preterm lung resulting in secondary insult via inflammatory mediated responses. Increased pro-inflammatory cytokines found in tracheal aspirates and blood samples from premature infants, including tumor necrosis factor alpha (TNFα), IL-8, IL-1β and IL-6, have been shown to correlate with increased risk of BPD [50,51,52].

### 2.4. Growth Restriction

Preterm infants that are small for gestational age (SGA) at birth or with intrauterine growth restriction (IUGR) are at increased risk for adverse pulmonary outcomes [53,54]. Studies have demonstrated a twofold increased risk of both BPD (28% vs. 14%) and neonatal mortality (23% vs. 11%) with SGA [54,55,56]. In addition, birthweight for gestational age is an important predictor of BPD-associated pulmonary hypertension [57]. While the association of growth restriction and BPD is in part secondary to compromised lung development, studies in bovine and murine IUGR models have demonstrated impacts on endothelial cell function, surfactant expression and inflammatory responses further influencing risks [58,59,60].

Extremely premature infants are at additional risk for postnatal growth restriction secondary to challenges of delivering optimal nutrition. Despite significant advances in the content and use of both enteral and parental nutritional support, 55% of infants born less than 27 weeks gestation demonstrate growth failure with weight less than 10th percentile at 36 weeks postmenstrual age [12,26,53]. Postnatal growth failure influences risks of developing BPD with data suggesting that delivery of adequate nutrition in the first week plays a critical role [7,53]. Studies have further identified that provision of optimal enteral feeding as compared to parenteral nutrition decreases risks of developing BPD [8]. Of interest are recent studies which demonstrate a decreased risk of BPD despite compromised growth with exclusive use of breast milk [61]. Despite evidence of normal alveolarization, murine models of postnatal growth restriction have identified pulmonary vascular remodeling, right ventricular hypertrophy and altered expression of key regulators of lung development including VEGF, HIF and mTOR, supporting the key contribution of postnatal nutrition to pulmonary vascular pathology and severity of disease [62].

### 2.5. Genetics

While BPD results from cumulative exposures to both the pre- and postnatal factors noted above, there is a growing interest in the heritable contributions to development of BPD. Twin studies provide insight into genetic predispositions as monozygotic twins share 100% of their genetic information while dizygotic twins are 50% concordant [63]. A total of 450 twin pairs were analyzed using mixed-effects logistic-regression and a latent variable probit model in a multicenter retrospective study. This analysis concluded that 65% of the variances in BPD susceptibility could be accounted for by genetic and shared environmental factors [64]. Subsequent multicenter studies confirmed the heritability of BPD by way of data identifying greater similarity in monozygotic as compared to dizygotic twins. One series of over 300 twins reported that genetics contributed to approximately 80% of the observed variance in rates of BPD [65].

More recently, several genome-wide association studies (GWAS) have been conducted to identify candidate single nucleotide polymorphisms associated with BPD. The largest evaluated over 1700 infants and failed to identify genomic loci or pathways that accounted for the previously described heritability for BPD [66]. A second, smaller analysis concluded that the SPOCK2 gene may represent a possible candidate susceptibility gene and a key regulator of alveolarization [67]. Rapid advances in genomics and proteomics suggest that regulators of susceptibility may eventually be identified, potentially allowing for targeted or individualized therapy to prevent and treat BPD.

## 3. Prevention of BPD

Management strategies are aimed at protecting against lung injury and the development of BPD. As the pathogenesis of disease is multifactorial, diverse approaches have been adopted including both ventilation and medical strategies. Interestingly, both antenatal steroids and surfactant reduce rates of RDS and improve survival; however, neither has been shown to reduce incidence of BPD [26].

### 3.1. Ventilation Strategies

Evidence of lung injury induced by volutrauma has led to efforts to promote “gentle ventilation,” in part through the use of permissive hypercapnia. However, the data to support this strategy has been inconsistent and long-term neurodevelopmental outcomes remain unknown [68,69]. Nonetheless, many units have adopted these practices, recognizing data supporting significant reduction in the need for mechanical ventilation with minimal liberalization of CO_2_ targets (>52 versus <48 mmHg) [70,71]. Gentle ventilation has also advocated for volume-targeted ventilation with meta-analysis to support this strategy in reducing rates of BPD and ventilator-associated inflammation [72,73,74]. Randomized clinical trials have demonstrated that both high-frequency jet (HFJV) and oscillatory ventilation (HFOV) have potential to reduce risk of BPD [75,76]; however, a Cochrane review of elective HFOV revealed only a small reduction in the incidence of chronic lung disease with notable inconsistency across the 19 studies included [77]. As many infants require more sedation with high frequency ventilation (HFV), prophylactic use of HFV remains controversial.

Significant efforts have been made to move away from use of invasive ventilation over the past two decades [26]. Meta-analysis of randomized clinical trials comparing prophylactic or early use of surfactant to initial support by continuous airway pressure (CPAP) have identified reduction in the combined outcome of death or BPD with avoidance of intubation [78,79,80]. These meta-analyses each included slightly different papers in their reviews, compiling data from studies using both prophylactic surfactant followed by rapid extubation (INSURE or INtubate, SURfactant, Extubate) and those which randomized to routine intubation. Despite variable studies included, the common finding of decreased risk of BPD strengthens recommendations for use of non-invasive strategies. As a result, many units are now opting for an early trial of CPAP to manage RDS.

Use of alternative non-invasive modalities including non-invasive positive pressure ventilation (NIPPV), bilevel nasal CPAP (biPAP) and high-flow nasal cannula (HFNC) is also increasing with some evidence to suggest these modes may also be effective in managing neonatal respiratory disease [81,82,83]. Single-center randomized trials have reported decreased need for intubation as well as reduced incidence of BPD with NIPPV as compared to continuous positive airway pressure (CPAP) [82]. In contrast, a Cochrane review including eight trials comparing NIPPV with CPAP identified less extubation failure with NIPPV, but no specific benefit with respect to risk of BPD [84]. This systematic review and research on NIPPV are challenged by highly variable clinical practices and management strategies. The optimal approach, impact on BPD and long-term outcomes with use of NIPPV remain to be defined [85].

Additional controversy exists surrounding the use of non-synchronized NIPPV. Synchronization can be achieved and has been described in premature infants using an abdominal pneumatic (or Graseby) capsule to detect diaphragmatic descent. This approach has the theoretical advantage of ensuring glottis patency before flow is triggered. While randomized trials using SNIPPV have not been performed, a retrospective study suggested comparable clinical outcomes, including rates of BPD, in infants managed with synchronized as compared to non-synchronized NIPPV [86]. More recently, some units have moved towards use of non-invasive ventilation with neurally adjusted ventilator assist (NAVA) to deliver synchronized breaths [87]. While this approach in theory may improve the patient-ventilator interaction and improve pulmonary outcomes, data to support this practice is still needed.

Finally, for those infants who do require intubation, an early trial of extubation is encouraged to potentially reduce risks of ventilator-induced lung injury. Studies have identified that an early attempt at extubation alone may decrease the risk of BPD, regardless of need for reintubation or duration of ventilation [88,89]. These provocative studies were both retrospective suggesting a need for additional prospective trials.

### 3.2. Saturation Targets

Extensive efforts have been made to define optimal saturation targets for premature infants with ongoing concerns for the quality of evidence available [90]. A meta-analysis including five separate trials did not identify differences in the outcomes of visual disability or BPD but raised concerns for increased risk of mortality (at 18–22 month CGA) with lower saturation targets (with low 85%–89% and high 91%–95%) as well as higher rates of necrotizing enterocolitis (NEC) [91,92].

Specific to the outcome of BPD, the Surfactant, Positive Pressure and Oxygenation Randomization Trial (SUPPORT) conducted in the US found slightly lower rates of BPD (38% vs. 41.7%) in the low-saturation group without statistical significance [93]. The Canadian Oxygen Trial (COT) similarly identified a similar trend with BPD rates of 31.8% and 33.1% in the low- and high-saturation groups, respectively [94]. Finally, the three trials conducted in New Zealand, the UK and Australia combined as the Benefits of Oxygen Saturation Trials (BOOST II) found similar trends with rates of 39.5% and 44.7% in the low- and high-saturation groups, again without statistical significance [95,96]. Use of higher saturation targets will inherently increase documented rates of BPD as infants cannot be weaned until they can maintain these higher goals. Nonetheless, the meta-analysis including all five studies still failed to identify a significant difference in rates of oxygen requirement at 36 weeks [92]. Despite the theoretical concerns for increased risk of oxidative lung injury and pulmonary vascular remodeling, many units now use higher saturation limits of 91%–95% based upon the collective finding of improved survival in these five trials. Some suggest that higher targets of 93%–97% should be considered with established BPD to reduce risks of subsequent pulmonary hypertension [97].

### 3.3. Corticosteroids

Postnatal glucocorticoids are recognized to reduce rates of BPD via reduced inflammation as well as the induction of lung maturational changes. However, the potential benefits of systemic steroids are often outweighed by concerns for long-term neurodevelopmental sequelae including increased risk of cerebral palsy (CP) [98,99]. Rates of use of systemic steroids for prevention of BPD have markedly decreased following American Academy of Pediatrics (AAP) and Canadian Pediatric Society (CPS) recommendations in 2002 against routine use [100,101]. Specifically, rates of postnatal corticosteroids use declined from 20% in 1997–2000 to only 12% in 2001–2002 and then again to 8% in 2004 with no significant change thereafter [26,102]. Concerns have been raised that a high-risk subpopulation may still benefit from lower dose and/or shorter courses of systemic steroids and that current practices have swung too far. Early or prophylactic use of low-dose courses of hydrocortisone in infants <28 weeks has been shown to reduce risk of BPD [103], with early (unpublished) data suggesting no adverse impact on neurodevelopmental outcomes at two years. Inhaled corticosteroids are used in some units as they are thought to be safer than systemic steroids; however, systematic reviews of inhalation corticosteroids have found inadequate data to support these practices [104,105].

In attempt to more reliably deliver corticosteroids directly to alveoli of infants at risk of early lung injury, co-administration of budesonide with surfactant has been considered. Yeh, et al. have reported significant decrease in the incidence of BPD or death in ventilated infants with severe RDS who received endotracheal budesonide [106]. Additional multicentered, randomized evaluation and follow-up studies of this practice are needed.

### 3.4. Caffeine

In the recent randomized, multicenter Caffeine for Apnea of Prematurity (CAP) trial, early initiation of caffeine was found to result in lower incidence of BPD as well as a shorter course of respiratory support as compared to controls [107]. The specific mechanism by which caffeine protects against lung injury remains unclear, and improved outcomes may have been due to decreased duration of ventilation alone. The benefits of caffeine were validated in additional cohorts and these data have collectively influenced practices in numerous units, including early initiation of caffeine in infants at risk of BPD [108,109].

### 3.5. Vitamin A

Vitamin A deficiency may predispose to chronic lung disease as it plays a critical role in maintaining the integrity of respiratory tract epithelium and is a key regulator of normal lung growth [110,111]. While meta-analysis suggests supplementation of preterm infants with vitamin A results in reduction in the combined outcome of death and BPD, the use of this therapy is highly variable as benefits were only observed in infants less than 1000 grams and the results were marginal [112]. Administration requires intramuscular injections which is associated with significant discomfort and potentially an increased risk of infection [113]. A large trial is currently in progress evaluating the efficacy of oral vitamin A supplementation with hopes that a simper route of administration may also be effective [114].

### 3.6. Nitric Oxide

Inhaled nitric oxide (iNO) for prevention of bronchopulmonary dysplasia deserves mention as it has been explored in numerous studies with inconsistent findings [115,116]. While a 24-day protocol initiated between 7 and 21 days was associated with decreased rates of BPD, combined evidence from the 14 randomized controlled trials in premature infants less than or equal to 34 weeks gestation showed equivocal effects on pulmonary morbidity, survival, and neurodevelopmental outcomes [117,118]. While this meta-analysis included highly variable protocols with respect to timing of initiation and dosing, the authors concluded that early use of iNO in preterm infants did not impact risk of brain injury or improve survival without BPD and that later use to prevent BPD might be effective but required further study. Subsequent individual-patient data meta-analysis suggested no specific benefit with prematurity and stated routine use could not be recommended [119]. Expert review of the topic resulted in consensus opinions from both the NIH and the American Academy of Pediatrics dissuading the routine use of iNO in premature infants [120,121]. Despite these statements, off-label use of iNO in extremely premature infants remains on the rise [122]. This may be due to use in grave circumstances where neonatologists feel that offering therapy is better than doing nothing [123]. There are also arguments that subpopulations may be more responsive and appropriate to consider for treatment, with data to suggest benefit after preterm premature rupture of membranes (PPROM) [122,124].

## 4. Long-Term Outcomes of BPD

Long-term outcomes of BPD remain difficult to characterize as adult populations currently available to study represent survivors of outdated care. While BPD tends to improve with ongoing lung development, data available from follow-up studies identify concerns for persistent pulmonary sequelae of disease. Notably, the impact on late pulmonary health as well as the consequences of additional infectious or environmental exposures remain poorly characterized.

It is important to recognize the tremendous emotional, medical, and financial efforts invested into care of extremely premature infants. The mean length of hospitalization for those born under 1000 grams is approximately 60 days with high rates of need for additional medical support including rehospitalization after discharge [125]. During their first year of life, 49% of infants with BPD will require rehospitalization and mortality risks associated with pulmonary complications of BPD are significant [126,127]. Retrospective studies identify survival rates of only 71%–81% with severe BPD requiring home ventilation [128,129]. Additional studies have shown that the incidence of sudden infant death syndrome was seven times greater in infants with BPD [130]. Follow-up studies of child and young adult survivors of BPD demonstrate concerns for compromised pulmonary function and defenses, asthma-like symptoms, pulmonary hypertension and exercise intolerance with altered responses to hypoxia.

### 4.1. Compromised Pulmonary Function

Numerous studies have evaluated long-term pulmonary function after premature birth alone. A meta-analysis including 59 articles identified that percent of predicted forced expiratory volume in 1 second (FEV1) is decreased in preterm-born survivors, even in patients who did not have a history of BPD [131]. Evaluation of teenaged or young adult survivors of BPD specifically confirmed lower FEV1, as well as decreased forced vital capacity (FVC) and forced expiratory flow rate at 50% of FVC as compared to controls [132,133,134]. Serial lung function testing of BPD infants at 6 months CGA and one year after identified compromised lung function in BPD infants (including decreased FVC, FEV at 0.5 seconds, and forced expiratory flows) without catchup during the study period [135]. This study further identified an association between growth in length and improvements of lung function, highlighting the important contribution of adequate nutrition in BPD survivors.

Additional studies have raised concerns for the persistence of pulmonary disease in patients with BPD. Correlation between maximal flow at functional residual capacity in infancy and forced expiratory flow at 8 years raises concerns that limited recovery occurs during late stages of alveolarization [136]. In addition, one longitudinal study of 8- and 18-year-old BPD survivors demonstrated deterioration of pulmonary function with age [134]. Airflow limitation has been attributed to dysanaptic growth, in which airways grow less rapidly than lung parenchyma resulting in fixed small airway obstruction [137]. High-resolution lung CT imaging obtained on young adult survivors confirmed that architectural distortion persists and correlates with compromised pulmonary function. However, the consequences of these findings on pulmonary disease in late adulthood remains unknown [138,139]. Of concern, animal studies of BPD which allow longer follow-up note that altered lung structure and compromised pulmonary function can persist into late adulthood [140].

### 4.2. Compromised Pulmonary Defenses

Exposure to environmental insults including respiratory infections, tobacco and pollution may complicate resolution of BPD and prolong risks of pulmonary morbidity [141,142]. Premature infants have increased susceptibility to infection which persists into childhood [143]. Common respiratory infections can result in severe morbidity and potential mortality in BPD survivors. Associated inflammation with these pulmonary insults may lead to additional lung injury in the already vulnerable recovering lung. Both epidemiologic data and animal models have demonstrated that BPD increases susceptibility to viral infection-induced lung injury with evidence of altered inflammation as a result of disrupted innate immunoregulatory pathways [144,145].

Beyond environmental insults, pulmonary morbidity in ex-preterm infants can be complicated by chronic reflux and microaspiration with risk of aspiration pneumonia and/or chronic inflammation. While the evidence is variable, improvement of respiratory status has been reported after Nissen fundoplication of infants with severe chronic lung disease with some programs advocating for this intervention in severe cases [146,147,148].

### 4.3. Asthma-Like Symptoms

Many survivors of BPD demonstrate a component of reactive airway disease. Long-term follow-up of infants born <26 weeks gestation identified that 25% had an asthma diagnosis at 11 years of age while over twice this percentage (56%) had evidence of abnormal spirometry [149]. While children with BPD have asthma-like symptoms, they are less likely to demonstrate airway hyper-responsiveness or response to bronchodilators as they may suffer a fixed peripheral airway narrowing [150]. In addition, co-morbid bronchomalacia or other central airway disease in ex-premature infants can result in exacerbated wheezing with use of bronchodilator therapy [151]. Although the evidence is limited with only one randomized trial [152], benefits from inhaled corticosteroids are also thought to be less consistent for children with BPD as compared to those with asthma [151]. One series suggests that outcomes of severe disease may be improved with use of chronic low-dose systemic steroids [148]. The reversibility and impact of this asthma-like phenotype on late adult pulmonary morbidity remains unknown.

### 4.4. Exercise Intolerance

Survivors of BPD may experience exacerbation of pulmonary morbidities with exercise or exposure to hypoxia. Significant risk of exercise-induced bronchoconstriction has been demonstrated in children with BPD consistent with concerns for reactive airway disease noted above [150]. However, in addition to risk of exercise-induced bronchoconstriction, BPD survivors are noted to have compromised gas exchange with physical activity. Treadmill exercise testing identify reduced gas transfer at rest and during activity which has been attributed to long-term derangements in lung structure or residual right ventricular dysfunction affecting cardiac output [153]. In addition, higher oxygen uptake during activity has been observed, which could contribute to early fatigability during prolonged exercise [154]. A recent detailed evaluation of 7 to 14-year-old survivors of BPD noted ventilatory limitation with exercise, including greater use of the ventilatory reserve, lower maximal ventilation and tidal volume. Peak exercise resulted in hypoxemia in 60% of BPD children with a concomitant increase in PaCO_2_ consistent with alveolar hypoventilation [155].

### 4.5. Abnormal Ventilatory Responses

Chemoreceptor function in preterm infants is dysmature, resulting in abnormal ventilatory responses to alterations of oxygen content in these patients. Specifically, normal responses of increased ventilation with hypoxia as well as decreased ventilation with hyperoxia may be altered. Persistent chemoreceptor dysfunction has been documented in survivors of BPD [156,157]. Inadequate response to hypoventilation and hypoxia may represent significant risk of morbidity in survivors of BPD who may also suffer co-morbid central airway disease or bronchomalacia. Abnormal respiratory muscle function in BPD survivors may further complicate this risk [156].

### 4.6. Pulmonary Arterial Hypertension

Dysmorphic pulmonary vasculature and compromised angiogenesis with BPD results in risk of elevated pulmonary pressures or BPD-associated pulmonary hypertension (PH). Retrospective studies of infants with BPD suggested that 25%–37% of infants with BPD develop associated PH, but these data are limited by inconsistent definition and screening protocols [21,22,23]. Evaluation of all oxygen-dependent infants by echocardiography at 36 weeks has become common in many units [13]. Identification of patients with elevated pulmonary pressures at earlier time points has been studied but remains of unclear benefit [158].

As previously noted, patients with BPD and elevated pulmonary pressures are at high risk for PH crisis and early mortality. Those who survive may ultimately demonstrate resolution of disease with additional lung growth. However, recent data suggests that subclinical right ventricular dysfunction can be detected in children assumed to have recovered from BPD-associated PH [159]. Longer-term outcomes in adulthood and risks of late pulmonary vascular disease with BPD-associated PH remain poorly defined. Animal models, which allow late follow-up, have raised concern for increased susceptibility to hypoxia-induced PH in adulthood [160].

While severity of BPD does not predict likelihood of associated pulmonary hypertension, sicker infants with prolonged ventilator and oxygen requirements are at greatest risk [158,161]. Additional risk factors include low gestational age, small for gestation age (SGA) status and oligohydramnious [21,22]. Even mild fetal growth restriction (<25th percentile) has been associated with PH [57], with evidence that decreased placental vascularity increases risk of disease [162]. Compromised lung growth with pulmonary vascular pruning may represent a relatively “fixed” component of PH, where abnormal vascular tone and vasoreactivity may be more responsive to therapy.

However, the threshold for intervention and optimal treatment of BPD-associated PH remains elusive with limited evidence to guide care [163]. While retrospective studies suggest improved echocardiographic evidence of PH with sildenafil, placebo-controlled studies in this patient population are lacking [164]. Use of vasodilator therapy is mostly guided by generalization from efficacy in idiopathic PH (IPH). Notably, the pathophysiology of vascular disease with BPD differs from IPH, with tendencies to improve over time and a significant component of vascular pruning rather than remodeling. Theoretical concerns for exacerbation of disease with vasodilator therapy exist, as treatment may further inhibit smooth muscle cell proliferation [165]. In addition, the relationship between pulmonary arterial pressure and disability are poorly defined, making the threshold to treatment difficult to define. Nonetheless, a recent large retrospective cohort study using data from the Pediatric Health Information System (PHIS) identified that 17% of the 598 infants diagnosed with BPD-associated PH received sildenafil. Likelihood of treatment correlated with gestation age, SGA and severity of BPD. Notably, this series identified a wide range to use with significant interinstitutional variation [166].

Some guidance for treatment has been provided in the literature. However, none of these to date are evidence based [163,167]. Kulik, et al. suggest that long-term drug therapy for PH should be reserved for patients with sound and reproducible evidence of pulmonary artery pressures at or near systemic levels in addition to reproducible evidence of right ventricular dysfunction by serial echocardiography. Other protocols have suggested that evidence of PH on echocardiogram (including pulmonary arterial pressures >25 mmHg, tricuspid velocity >2.8 m/s, or septal curvature/paradoxical motion) should be an indication for cardiac catheterization with vasoreactivity testing prior to initiation of treatment [167]. However, clinical stability and/or access to this procedure may require modification with initiation of medications prior to catheterization. These authors suggest that confirmation of PH by catheterization would be an indication for therapy, while the specific choice of medication should be influenced by vasoreactivity tests. Specifically, they suggest calcium-channel inhibitors should be considered with a positive test and sildenafil followed by bosentan with lack of vasoreactivity. In a retrospective analysis of infants with ventilator-dependent BPD, Gien, et al. implied that PA pressures greater than half systemic should be a consideration for cardiac catheterization with vasoreactivity testing [148]. The authors further highlighted that identification of pulmonary vein stenosis, aortopulmonary collaterals or LV diastolic dysfunction may influence care as vasodilator therapy in these settings would result in increased pulmonary blood flow and risk of pulmonary edema. Afterload reduction, including milrinone initially, followed by angiotensin-converting enzyme inhibitors, was suggested. Pulmonary vasodilation acutely with nitric oxide followed by transition to sildenafil and as-needed bosentan was suggested in all other PH cases.

While the past decade has been notable for increasing use of vasodilator therapy in the treatment of BPD-associated PH [166], randomized controlled trials are much in need to clarify the optimal use of these therapies with BPD associated PH.

## 5. Conclusions

The definition, pathophysiology, and management of bronchopulmonary dysplasia (BPD) has evolved significantly since first described by Northway almost 50 years ago. Advances in neonatal care have resulted in increased rates of survival of extremely premature infants leading to both a new set of management challenges as well as an emerging population of long-term survivors of BPD. Interdisciplinary care to address the complex pulmonary, nutritional and developmental needs of these patients is critical and may itself influence outcomes of severe BPD [148].

Randomized therapeutic studies in addition to longitudinal evaluation of these patients remains essential to optimize care and further discern risk factors for morbidity. While these studies require significant resources, they are much in need as evidence for optimal treatment is lacking. In addition, little is known regarding pulmonary outcomes of BPD beyond the second decade of life. Of concern are data highlighting the potential for increased risk of subclinical right ventricular dysfunction, obstructive lung disease, exercise intolerance, and asthma-like symptoms in survivors. Abnormal response to hypoxia and central airway disease may further exacerbate illness with risks of sleep disordered breathing. These baseline morbidities, complicated by environmental and infectious exposures, may represent a significant challenge for the aging cohort of BPD survivors.

As trends demonstrate increasing survival of extremely premature infants, nearly half of whom will be diagnosed with BPD, it is imperative that future studies investigate mechanisms and risk factors influencing long-term morbidity with an overall goal of reducing the burden of disease.

## Figures and Tables

**Table 1:** Definition of Bronchopulmonary Dysplasia: Diagnostic Criteria. Reprinted with permission of the American Thoracic Society. Copyright © 2016 American Thoracic Society. Jobe, A.H.; Bancalari, E. Bronchopulmonary Dysplasia. *Am. J. Respir. Crit. Care Med.* 2001, 163, 1723–1729. The *American Journal of Respiratory and Critical Care Medicine* is an official journal of the American Thoracic Society.

Gestational Age	<32 wk	≥32 wk
Time point of assessment	36 wk PMA or discharge to home, whichever comes first	>28 d but <56 d postnatal age or discharge to home, whichever comes first
	Treatment with oxygen > 21% for at least 28 d **plus**	
Mild BPD	Breathing room air at 36 wk PMA or discharge, whichever comes first	Breathing room air by 56 d postnatal age or discharge, whichever comes first
Moderate BPD	Need * for <30% oxygen at 36 wk PMA or discharge, whichever comes first	Need * for <30% oxygen at 56 d postnatal age or discharge, whichever comes first
Severe BPD	Need * for ≥30% oxygen and/or positive pressure, (PPV or NCPAP) at 36 wk PMA or discharge, whichever comes first	Need * for ≥30% oxygen and/or positive pressure (PPV or NCPAP) at 56 d postnatal age or discharge, whichever comes first

Definition of abbreviations: BPD = bronchopulmonary dysplasia; NCPAP = nasal continuous positive airway pressure; PMA = post menstrual age; PPV = positive pressure ventilation. * A physiologic test confirming that the oxygen requirement at the assessment time point remains to be defined. This assessment may include a pulse oximetry saturation range.

BPD usually develops in neonates being treated with oxygen and positive pressure ventilation for respiratory failure, most commonly respiratory (e.g., central apnea or diaphragmatic paralysis) do not have BPD unless they also develop parenchymal lung disease and exhibit clinical feature of respiratory distress. A day of treatment with oxygen > 21% means that the infant received oxygen > 21% for more than 12 h on that day. Treatment with oxygen > 21% and/or positive pressure at 36 wk PMA, or at 56 d postnatal age or discharge, should not reflect an “acute” event, but should rather reflect the infant’s usual daily therapy for several days preceding and following 36 wk PMA, 56 d postnatal age, or discharge.
